# Mendelian susceptibility to mycobacterial infection should be ruled out in *Mycobacterium chelonae* empyema

**DOI:** 10.4103/1817-1737.58964

**Published:** 2010

**Authors:** Luis Ignacio González-Granado

**Affiliations:** *Immunodeficiencies Unit, Hospital 12 Octubre, Carretera Andalucia km 5,400, Madrid, Spain. E-mail: nachgonzalez@gmail.com*

Sir,

I am grateful to Wali S[1] for his contribution to the knowledge of nontuberculous mycobacterial infections.[1] Nevertheless, I would like to make one comment - When assessing immunocompetence, we need to ask ourselves what are we looking for. It is well known that patients with deficiency of interferon gamma1/2R-IL12/R-IL23/R pathway (also known as Mendelian susceptibility to mycobacterial disease (MSMD) are prone to non tuberculous mycobacterial infections. Patients with defects in the interferon gamma pathway are predisposed to mycobacterial diseases, while those with defects in the IL-12 pathway are frequently threatened by nontyphoid (systemic) salmonellosis. Tuberculosis has been described in both of these signaling pathway defects. These disorders are genetically different but immunologically similar as impaired IFNγ-mediated immunity is the common pathogenic mechanism accounting for mycobacterial infection in all patients. The severity of the histological and clinical phenotype depends on the type of genetic defect. Genetic dissection of the IFNgamma/IL-12/IL23 pathway has improved our understanding of the human immune response to mycobacteria in the last ten years and help us to elucidate the genetic bases of tuberculosis.[2,3] This assessment has not been done in any of the two cases of *M. chelonae* empyema reported,[4] and it could elucidate the immunocompromissed status against mycobacterial infections.
